# An Instructive Lesson

**Published:** 1891-01

**Authors:** J. H. Jackson


### AN INSTRUCTIVE LESSON.

J. H. JACKSON, M. D.

Mr. M——, aged twenty-nine, dark complexion, black eyes and hair; a strong, robust man, consulted me about an acute attack of gonorrhœa.

The prepuce was infiltrated and considerably distended, particularly on right side; many painful erections at night; profuse discharge of greenish-yellow matter; burning for about half an inch of the anterior portion of urethra; very frequent and profuse urination, with inability to retain urine if the desire is not immediately gratified.

The œdema of prepuce might call for Natrum sulphuricum, Rhus-tox., Apis, or in fact any remedy.

Several remedies cover the urgent and profuse urination, but the question is, What remedy covers the man's individuality?

His pulse was very full, it felt quite as large as the largest round lead-pencil.

The carotids were seen to pulsate quite plainly; the eyes were brilliant and he shunned the light.

It was quite plain to me that Belladonna was the most suitable medicament for this case of sickness, so Belladonna was given in the CM potency. First one dose and then Sac-lac. every three hours. In five days there was no perceptible change for the better, and Belladonna was given three times a day. The only improvement noticeable after a week was improvement in pulse and in the pulsation of carotids.

Sulphur CM controlled the urging and inability to hold urine, but the œdema of prepuce, discharge, erections, and pains were unchanged. A new symptom appeared, swelling of inguinal glands, particularly on right side, and the discharge was more greenish. Merc-sol. CM in a single dose and in repeated doses did not seem to change the case for the better. So Medorrhinum MM and MMM were given, and all symptoms were about the same, though two weeks were given for it to act.

Methinks I have somewhere read, or have heard, that some brilliant man has at sometime said, that Merc-cor., one part to five thousand of water would kill some invisible beast in the urethra, and by its use we would not get "left" in the race. Perhaps I dreamed this, for how could a professional homœopath recommend such folly to other men who had chosen to be guided by a *law* of nature in their efforts to heal the sick?

Well, the case under consideration demanded Belladonna.

Remedies against psora were given, principally Sulphur, and I will here mention a symptom that as a guide to the choice of Sulphur, has *never* failed to be a true guide.

Patients will go to sleep on either side, and *when they awake they will invariably find themselves on their back.*

Several remedies have the symptom "sleeps on back," but I know of no remedy but Sulphur that covers the symptom he invariably finds that *during* sleep he has turned from position on side to position on back. This may occur twenty times a night.

And to me this is quite as characteristic of Sulphur as sleeping into distress is of Lachesis.

I treated this man for six months with Belladonna CM and 10 MM with a slow improvement of his symptoms. The painful erections were gone; the discharge was greatly diminished, and the œdema of prepuce nearly gone, but yet he was not cured. A slight cold or a cup of coffee or beer and back would come an aggravation of his complaint.

What a battle of words and argumentation to keep this man from violating the law of metastasis by injections!

If any one should offer me a cash fee of like amount to do the same amount of talking, I would think it small pay for so great a labor.

I knew that at some time he had suffered a suppression of some complaint by improper treatment, but he could not, or would not answer my frequent questions in regard to past sicknesses. He would invariably say, "I have never had a doctor for anything."

But here was Belladonna plainly indicated. Was the law of the similars a lie? Was this a case for violent and senseless local treatment?

No, there was somewhere in this man's history a maltreatment of some complaint, and its suppression had added a morbific force that was acting in conjunction with the dynamis of gonorhœal contamination. I was certain of it, from past experience.

The instructive lesson of confirmation came. One morning the patient entered my office and said, "I have a very sore throat." I looked at it and both tonsils, and to some extent the walls of pharynx and soft palate were covered by a diphtheritic membrane. My constant admonition against local treatment had not been without avail, for he said, "Doctor, you have said so much about local applications that I was afraid to use my usual gargle of Chlorate of Potash, that on four previous occasions has cured (?) just such a throat as this."

I asked him how about the discharge from penis. He replied, "*Isn't it funny, since my throat got sore that discharge has entirely left.*"

Belladonna CM one dose, and quiet in the house for four days and that was the end of all his symptoms.

